# The Voltage-Gated Sodium Channel Na_v_1.8 Is Expressed in Human Sperm

**DOI:** 10.1371/journal.pone.0076084

**Published:** 2013-09-27

**Authors:** Antonio Cejudo-Roman, Francisco M. Pinto, Nerea Subirán, Cristina G. Ravina, Manuel Fernández-Sánchez, Natalia Pérez-Hernández, Ricardo Pérez, Alberto Pacheco, Jon Irazusta, Luz Candenas

**Affiliations:** 1 Instituto de Investigaciones Químicas (IIQ), CSIC - Universidad de Sevilla, Sevilla, Spain; 2 Departamento de Fisiología, Facultad de Medicina y Odontología, Universidad del País Vasco, Leioa, Bizkaia, Spain; 3 Instituto Valenciano de Infertilidad, Sevilla, Spain; 4 Instituto Madrileño de Infertilidad, Madrid, Spain; Cinvestav-IPN, Mexico

## Abstract

The role of Na^+^ fluxes through voltage-gated sodium channels in the regulation of sperm cell function remains poorly understood. Previously, we reported that several genes encoding voltage-gated Na^+^ channels were expressed in human testis and mature spermatozoa. In this study, we analyzed the presence and function of the TTX-resistant VGSC α subunit Nav1.8 in human capacitated sperm cells. Using an RT-PCR assay, we found that the mRNA of the gene *SCN10A*, that encode Na _v_1.8, was abundantly and specifically expressed in human testis and ejaculated spermatozoa. The Na _v_1.8 protein was detected in capacitated sperm cells using three different specific antibodies against this channel. Positive immunoreactivity was mainly located in the neck and the principal piece of the flagellum. The presence of Na _v_1.8 in sperm cells was confirmed by Western blot. Functional studies demonstrated that the increases in progressive motility produced by veratridine, a voltage-gated sodium channel activator, were reduced in sperm cells preincubated with TTX (10 μM), the Na _v_1.8 antagonist A-803467, or a specific Na _v_1.8 antibody. Veratridine elicited similar percentage increases in progressive motility in sperm cells maintained in Ca^2+^-containing or Ca^2+^-free solution and did not induce hyperactivation or the acrosome reaction. Veratridine caused a rise in sperm intracellular Na^+^, [Na^+^]_i_, and the sustained phase of the response was inhibited in the presence of A-803467. These results verify that the Na^+^ channel Na _v_1.8 is present in human sperm cells and demonstrate that this channel participates in the regulation of sperm function.

## Introduction

Ion channels play a central role in the regulation of sperm intra- and inter-cellular signaling [1-9]. The rapid ion fluxes through these membrane proteins permit a quick transfer of information between sperm and its surrounding [1,10-12]. This communication is essential for correct sperm guidance throughout the female reproductive tract as well as for acquisition of fertilization competence and interaction with the oocyte [10-13]. Many different ion channels have been identified in the sperm cell membrane. Among them, Ca^2+^, K^+^, H^+^ and anion channels are widely distributed in the head and flagellum and play an important role in regulating sperm function including motility, capacitation and acrosome reaction [1,2,5,6,11,12]. Recently, different works have pointed to the importance of the sperm-specific Ca^2+^ channels CatSper in the control of intracellular Ca^2+^ concentration, [Ca^2+^]_i_, in sperm cells [3,7,8,14,15]. These channels mediate the progesterone-induced Ca^2+^ influx in human sperm and are essential for sperm function and male fertility [7,8,15]. Na^+^ channels should also play an important role in sperm, as the gradient of this ion across the plasma membrane plays a central role in the regulation of membrane potential (E_m_), a parameter that govern the rate and direction of ion-flow through channels and exchangers and modulates the intracellular pH (pH_i_) [16,17]. In this context, the presence of epithelial Na^+^ channels of the ENaC family [17] and of voltage-gated Na^+^ channels (VGSC) [18] has been demonstrated in sperm cells, although their role remain poorly understood.

VGSCs are complex proteins composed by a α and one or more β auxiliary subunits [19]. Nine different VGSCs α subunits have been cloned in mammals, each one encoded by a different gene [19,20]. They can be further characterized by their sensitivity to the highly selective blocker tetrodotoxin (TTX). The TTX-sensitive α subunits are inhibited in the nanomolar range by TTX and include Na _v_1.1, Na _v_1.2, Na _v_1.3, Na _v_1.4, Na _v_1.6 and Na _v_1.7. The TTX-resistant α subunits are inhibited in the micromolar range by TTX and include Na _v_1.5, Na_v_1.8 and Na _v_1.9 [19-21].

We have recently reported that the mRNAs that encode the different Na_v_ α subunits (Na _v_1.1-1.9) are expressed in human spermatozoa [18]. Immunofluorescence studies showed that, with the exception of Na_v_1.1 and Na_v_1.3, the Na_v_ channel proteins were present in sperm cells and show specific and different localizations [18]. This is particularly interesting in the case of Na_v_1.8, as it is currently accepted that its expression is restricted to certain neuronal populations present in the dorsal root ganglia (DRG), the heart and the retina [22-24]. Na _v_1.8 participates in the transmission of pain signals induced by cold, heat and mechanical stimuli [22,25] and, as reported very recently, has a direct modulatory role in cardiac electrophysiology [24]. The possible role of Na _v_1.8 in the regulation of mammalian fertility has not been studied. In the present study, we aimed to characterize the presence and function of the Na _v_1.8 sodium channel in capacitated human sperm.

## Materials and Methods

### Ethics Statement

This study was approved by the Ethic Committee of Consejo Superior de Investigaciones Científicas (CSIC, Spain) and all donors gave written informed consent.

### Semen samples and sperm preparation

Freshly ejaculated semen was collected from 148 healthy donors (18-35 years old) after 3-4 days sexual abstinence and examined following WHO guidelines [26]. The semen parameters (total fluid volume, sperm concentration, motility and morphology) of all samples fell within WHO normality criteria. Samples were allowed to liquefy at 37°C, washed with modified human tubal fluid (mHTF, Irvine Scientific, Santa Ana, USA) supplemented with 10 mM HEPES and 0.5% BSA and processed as previously described [27]. Briefly, spermatozoa were separated from seminal plasma by a Percoll discontinuous density gradient (Spermgrad-125, Vitrolife, Kungsbacka, Sweden), allowed to swim-up for 1 h at 37°C and the supernatant carefully aspirated. Spermatozoa were capacitated by incubation for 2 h at 37°C in 5% CO_2_ for subsequent experiments.

### RNA extraction and real-time quantitative polymerase chain reaction (qPCR)

The expression of *SCN10*, the gene that encodes Na _v_1.8, was analyzed in RNAs from 20 different human tissues (Human total RNA master panel, BD-Biosciences, Clontech, Palo Alto, CA). In addition, total RNA was extracted from human sperm pools, each containing sperm from 2 different donors, using TriReagent (Sigma, St. Louis, MO). Complementary DNAs (cDNAs) were synthesized using the Quantitect Reverse Transcription kit (Qiagen, Venlo, The Netherlands). The cDNA samples were then amplified by PCR using specific oligonucleotide primers designed with the software Primer 3 and synthesized by Sigma-Genosys (Cambridge, UK). Two different intron-spanning primer pairs were designed to amplify human *SCN10A* and their sequences were: pair (1), forward 5´-ATGACCCGAACTGACCTTCC-3´ and reverse 5´-TGACGCTAAAATCCAGCC AGT-3´, giving a PCR product of 151 bp; pair (2), forward 5´-TGACAGAGGAGCAGAAGAAATACTACA-3´ and reverse 5´-GTTGAGGCAGATGAGGACCA-3´, giving a PCR product of 164 bp. The genes encoding β-actin (*ACTB*); protein phosphatase-1 catalytic subunit beta-isoform (*PPP1CB*), glyceraldehyde-3-phosphate-dehydrogenase (*GAPDH*) and polymerase (RNA) II (DNA directed) polypeptide A (*POLR2A*) were chosen as housekeeping genes for normalizing the PCR data on the basis of previous studies on human testis and human sperm [18,21].

End-point PCR was performed for 30, 35, 40 or 45 cycles and used to assess the specificity of the amplified transcripts. The PCR products were separated by electrophoresis in agarose gel stained with ethidium bromide and the amplicon sizes were verified by comparison with a DNA mass ladder. Real-time qPCR was used to quantify the expression of *SCN10* in human tissues, which was carried out by the 2^-∆∆C^_T_ method, as described previously [21,28]. qPCR was performed on a Bio-Rad iCycler iQ real-time detection apparatus (Bio-Rad Laboratories, Hercules, CA) using a FastStart SYBR Green Master (Roche Diagnostics GmbH, Manheim, Germany). The parameters of PCR amplification were: 10 sec at 94°C, 20 sec at 60°C and 30 seconds at 72°C, for 50 cycles. The identity of each product was established by DNA sequence analysis and the specificity of PCR reactions was confirmed by melting curve analysis of the products and by size verification of the amplicon in a conventional agarose gel.

The fold change of the target gene expression was expressed relative to the geometric mean mRNA expression of the housekeeping genes in each sample, as described by Vandesompele et al. [29]. Each assay was performed in triplicate and three negative controls were run for each assay: no template, no reverse transcriptase and no RNA in the reverse transcriptase reaction.

### Flow Cytometry

Spermatozoa were adjusted to a concentration of 25 x 10^6^ cells/ml, fixed in paraformaldehyde 4% during 10 min and permeabilized in 0.5% Triton X-100 for 30 min. Cells were washed twice in PBS at 400 g for 5 min and incubated in blocking medium (PBS with 2% casein) for 120 min. Samples were incubated overnight at 4 °C with a primary antibody designed to recognize human Na _v_1.8 (rabbit polyclonal ab-66743, rabbit polyclonal ab-83936 or mouse monoclonal ab-93616, all from Abcam, Cambridge, UK). These primary antibodies were diluted 1:200 in PBS and incubated overnight at 4°C. A goat anti-rabbit (for ab-66743 or ab-83936) or goat anti-mouse (for ab-93616) IgG (Santa Cruz Biotechnology, Santa Cruz, CA) was used as secondary antibody at a 1:200 dilution and nuclei were stained with 0.2 μg/ml propidium iodide (PI). Negative controls were performed omitting the primary antibody before secondary antibody addition. Data from at least 10,000 events were captured on a BD Accuri C6 flow cytometer (BD Biosciences, San José, CA) and FITC and PI fluorescence were analyzed with CFlow Plus software.

### Immunofluorescence

Capacitated sperm cells were washed, resuspended in PBS and smeared onto poli-L-lysine-coated slides. Spermatozoa were then fixed by incubation in cold methanol (-20°C) for 20 min. After blocking for 120 min with 2% casein in PBS, test slides were incubated overnight at 4°C with rabbit (ab-66743, ab-83936) or mouse (ab-93616) anti-Na _v_1.8 (dilution 1:200). Negative control slides were not exposed to the primary antibody and were incubated in PBS in the same conditions as the test slides. Samples were extensively washed and incubated for 60 min with appropriate FITC-conjugated secondary antibodies. Slides were further washed in PBS, mounted using Prolong Gold antifade reagent with DAPI (Invitrogen, Molecular Probes, Eugene, OR) and examined with a Olympus BX-51 fluorescence microscopy (Tokyo, Japan) using a 60x immersion objective.

### Western Blot experiments

Total proteins were extracted from sperm cells as described previously [27,30]. The protein content was quantified using a bicinchoninic acid (BCA) protein assay kit (Pierce, Rockford, IL) and 40 μg sperm protein were loaded on 10% sodium dodecyl sulphate (SDS)-PAGE gels. Proteins were separated by electrophoresis, transferred to polyvinyldifluoride (PVDF) membranes and incubated with an anti-Na _v_1.8 antibody (ab-66743, ab-83936 or ab-93616). Immunoreactivity was detected by treatment with appropriate HRP-conjugated secondary antibody and developed with the Amersham advance enhanced chemiluminescence (ECL) kit (Buckinghamshire, UK). Primary antibody dilution was 1:2000 and for the secondary antibody it was 1:50000.

To analyze phosphorylation of sperm proteins on tyrosine residues, membranes were incubated with a mouse monoclonal antibody against human phosphotyrosine (pY20, SC-508, Sta. Cruz) and tyrosine phosphorylation was immunodetected by treatment with HRP-conjugated secondary mouse antibody. Experimental conditions were similar to those described above using a 1:5000 primary antibody dilution and a 1:20000 secondary antibody dilution.

### Sperm motility studies

Spermatozoa were capacitated and adjusted to a concentration of 50 x 10^6^ cells/ml. Motility analysis was conducted by computer-assisted sperm analysis (CASA) (Sperm Class Analyzer, S.C.A., Microptic, Barcelona, Spain) as described previously [18,30]. The following kinematics parameters were measured: curvilinear velocity (VCL, μm/s); straight-line velocity (VSL, μm/s), average-path velocity (VAP, μm/s); amplitude of lateral head displacement (ALH, μm); linearity of progression (LIN=VSL/VCL x 100); straightness (STR=VSL/VAP x 100); motility and hyperactivated motility. The motility pattern of sperm samples was established following WHO guidelines [26] and defined as: "A" grade sperm (rapid progressive motility), "B" grade (slow progressive motility), “C” grade (non-progressive motility) and “D” grade (immobile). Progressive motility (*A* + *B*), non-progressive motility (*C*) and immotility (D) were measured as a percentage of the total (*A+B+C+D*) that was considered as 100%.

To investigate the effects of drugs, individual sperm samples were divided in several aliquots and each aliquot was treated with a single concentration of veratridine (10 μM) (Sigma) or the corresponding solvent (time-matched paired controls). Sperm motility was measured 5 min before veratridine addition (initial value) and after a contact time of 2, 15, 30, 60, 120, 180 and 240 min. In parallel experiments, the effect of veratridine or its solvent was investigated in aliquots pretreated during the last 15 min of capacitation with the specific Na _v_1.8 antagonist A-803467 (Sigma) (1 μM) [25] or during capacitation (2 h) with TTX (Alomone Labs, Jerusalem, Israel)) (0.01 or 10 μM), the Na _v_1.8 antibody ab-66743 (dilution 1:50) or the corresponding solvent. The concentrations and times of incubation with the different drugs were chosen from previous experiments ( [18] unpublished data). Additional experiments were performed in similar conditions to evaluate the effects of TTX (10 μM), A-803467 (1 μM), the Na _v_1.8 antibody ab-66743 (dilution 1:50) or the corresponding solvent on normal sperm motility in the absence of veratridine or its solvent.

In another set of experiments, the effects of veratridine or its solvent were analyzed in a Ca^2+^-free medium, prepared by omitting Ca^2+^ from mHTF. In these conditions, the Ca^2+^ concentration in the physiological solution was in the μM order [9].

Values of sperm progressive motility, non-progressive motility and immotility were expressed as the positive or negative percentage increment in motility produced by the drug relative to the value observed at the same time in solvent-treated time-matched paired controls (Δ sperm motility).

### Acrosome reaction assays

Acrosomal status was assessed following previously described procedures [30,31]. Briefly, capacitated sperm aliquots (10 x 10^6^ cells/ml) were untreated (time-matched paired controls) or treated for different times with veratridine (10 μM), the ionophore A23187 (10 μM) or the corresponding solvent at 37°C, 5% CO_2_. Sperm cells were fixed/permeabilized in ice-cold methanol, stained with fluorescein isothyocianate-conjugated lectin from *Pisum sativum* (FITC-PSA, Sigma) and the acrosomal status evaluated by fluorescence microscopy. Spermatozoa displaying an intact acrosome are strongly labeled with FITC-PSA at the acrosomal region whereas AR reacted cells show no labeling in this region with or without labeling of the equatorial region. Percentage AR values were calculated by the formula: (%AR reacted spermatozoa in treated aliquots)-(%AR reacted spermatozoa in the corresponding solvent-treated aliquots).

### Measurements of sperm intracellular Ca^2+^ and Na^+^

For measurement of intracellular Ca^2+^, [Ca^2+^]_i_, capacitated spermatozoa were adjusted to a concentration of 10 x 10^6^ cells/ml and incubated with the acetoxymethyl ester form of Fura-2 (Fura-2/AM, 8 μM, Molecular Probes, Invitrogen, Eugene, OR, USA) for 60 min at room temperature in the presence of the noncytotoxic detergent pluronic acid (0.1%, Molecular Probes). After loading, the cells were washed, resuspended in mHTF solution with or without Ca^2+^ and used within the next 2–7 hours, following previously described procedures [30]. Sperm aliquots (1 ml) were placed in the quartz cuvette of a spectrofluorometer (SLM Aminco-Bowman, Series 2, Microbeam, Barcelona, Spain) and magnetically stirred at 37°C. The emitted fluorescence was measured at 510 nm. Changes in [Ca^2+^]_i_ were monitored using the Fura-2 (F340/F380) fluorescence ratio as previously described [30].

The effects of veratridine were studied on sperm aliquots untreated or pretreated for 6 h with the Na _v_1.8 antibody ab-66743 (dilution 1:50) or for 15 min with A-803467 (1 μM), TTX (10 μM) or the corresponding solvent. Progesterone (1 μM) was added to the cuvette at the end of the experiment and served as an internal control. Calibration of [Ca^2+^]_i_ was achieved according to the equation of Grynkiewicz et al. [32] adding Triton X-100 (5%), to obtain the maximal response, followed by addition of EGTA (40 mM) to obtain the minimal response.

To analyze the effect of veratridine on sperm intracellular Na^+^ concentration, [Na^+^]_i_, capacitated spermatozoa were adjusted to a concentration of 35 x 10^6^ cells/ml and incubated in mHTF with the acetoxymethyl ester form of SBFI (SBFI⁄AM, 10 µm; Invitrogen) for 360 min at room temperature in the presence of pluronic acid (0.1%). After loading, the cells were washed and resuspended in mHTF. In some experiments Na^+^ was omitted from mHTF and replaced iso-osmotically with choline chloride. Spermatozoa aliquots (1 ml) were then placed in the cuvette of the spectrofluorometer and processed as described above for the Fura-2-loaded samples. The sperm suspension was alternatively illuminated with two excitation wavelengths (340 nm and 385 nm) and the emitted fluorescence was measured at 495 nm. Changes in [Na^+^]_i_ were monitored and expressed using the SBFI (F340⁄ F385) fluorescence ratio. As an internal control, the response upon addition of EGTA (4 mM) was monitored at the end of the experiment [33]. The effects of veratridine were studied in sperm aliquots untreated or pretreated for 15 min with A-803467 (1 μM) or its solvent.

### Statistical analysis

Values (means ± SEM) were obtained by pooling individual data. Unless otherwise indicated, *n* represents the number of experiments in sperm samples from *n* different donors. Multiple means were compared by one-way analysis of variance (ANOVA) followed by Dunnett’s multiple comparison test and Student’s *t*-test was used to compare the means of two groups. These procedures were undertaken using GraphPad PRISM (version 5.0) program. A value of *P*<0.05 was considered significant.

## Results

### Analysis of sperm capacitation

Sperm capacitation was determined by analyzing phosphorylation of sperm proteins on tyrosine residues at different times of incubation at 37°C in 5% CO_2_. Tyrosine phosphorylation can be observed after 1 h incubation and was similar in sperm preparations maintained for 2 h or 4 h in the CO_2_ incubator (Figure S1). A time of 2 h was used for capacitation in subsequent experiments.

### mRNA expression of *SCN10A* in human tissues and spermatozoa

We previously found, using end-point RT-PCR, that *SCN10A* was expressed in human testis and sperm cells [18,21]. In this study, we designed two additional primer pairs and used real-time qPCR to quantify the mRNA levels of *SCN10A* in cDNA from 20 different human tissues.

**Figure 1:**
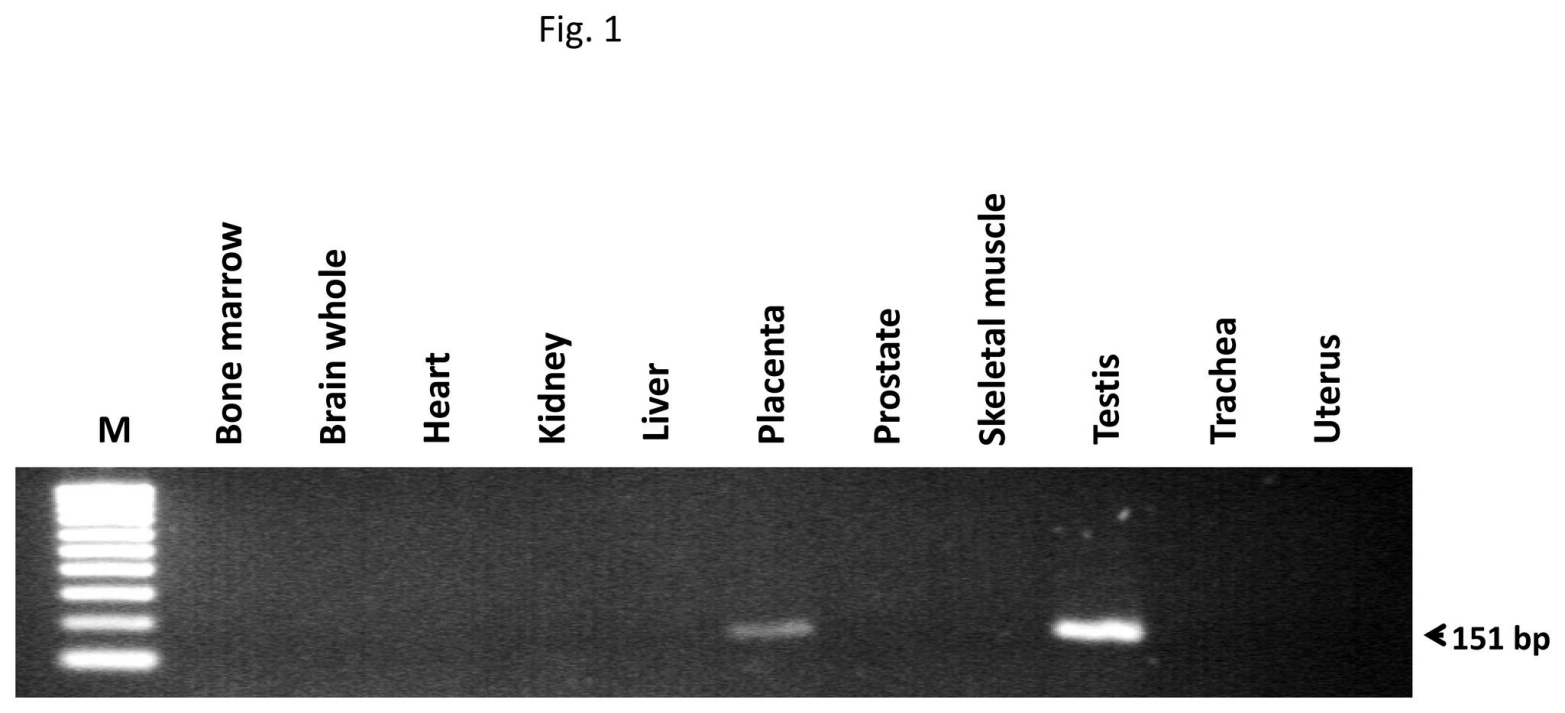
Expression of *SCN10A*, the gene that encode Na _v_1.8, in different human tissues. After 35 cycles of PCR amplification, the specific band corresponding to *SCN10A* was detected in human testis and placenta. M, molecular size standards. The figure shows a representative RT-PCR experiment, *n* = 3.

After PCR amplification for 30 cycles with any of the specific primer pairs, the *SCN10A* mRNA was only detected in human testis. After amplification for 35 cycles, the specific transcript was also detected in the placenta (Figure 1) and appeared in the trachea after amplification for 40 cycles. Real-time qPCR demonstrated that, in comparison with human testis, the mRNA levels were 5.1 ± 0.9 and 52.0 ± 10.8 fold lower in the placenta and the trachea, respectively (*n*= 3, *P*<0.05, Student’s *t* test).

After 45 cycles of PCR amplification, a low *SCN10A* expression was observed in eight additional tissues: the bone marrow, the brain, the heart, the kidney, the liver, the prostate, the uterus and the skeletal muscle. In each of these human tissues, the *SCN10A* expression was more than 100-fold lower than in the human testis (Figure 1). No fragment amplification was detected in the negative control samples (not shown).

The *SCN10A* transcript was present in cDNA from human sperm and was detected with the two specific primer pairs in all samples assayed (*n* = 8 different sperm samples, with each sample being a pool of sperm from two different donors, data not shown).

### Immunodetection of the Nav1.8 channel protein in human spermatozoa

**Figure 2:**
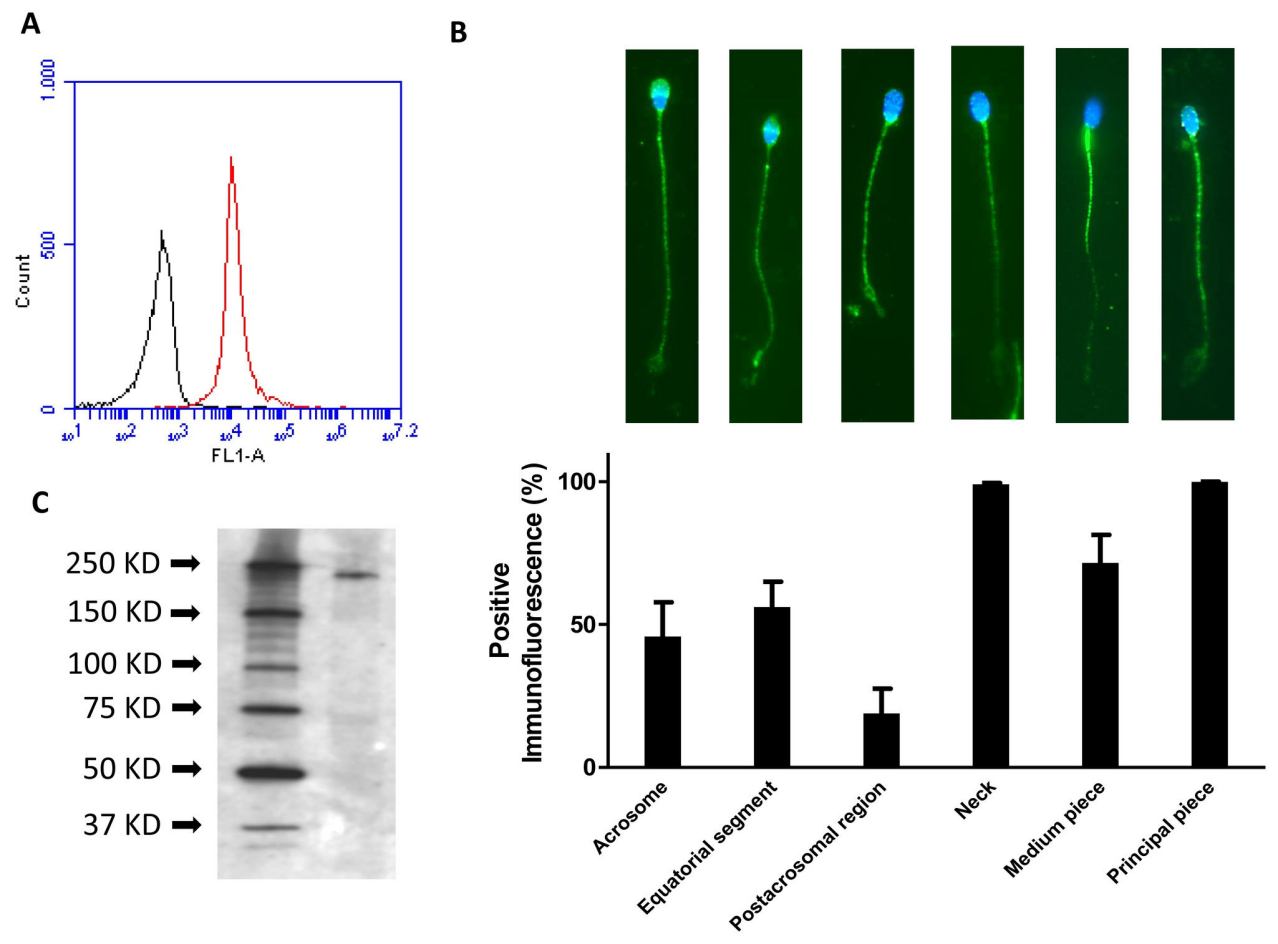
Immunolocalization of the Nav1.8 protein in human sperm. (A) Flow cytometry plots of spermatozoa after overnight labeling with a Na _v_1.8 antibody (red plot) and the negative control treated with secondary antibody alone (black plot), *n* = 9. (B) Immunofluorescence images of sperm cells stained with a primary antibody against Na _v_1.8. Bar graph represents the distribution of this voltage-gated sodium channel in different sperm regions and the percentage of sperm cells showing each specific localization, *n* = 9. Scale bar, 10 μM. (C) Western blot analysis of Nav1.8 in human sperm homogenates using the Nav1.8 rabbit polyclonal antibody ab-66743. Molecular mass is indicated on the right side of the panel. The figure is representative of results obtained in 6 separate protein preparations from 6 different donors.

Flow cytometry analyses were carried out using three different specific Na _v_1.8 antibodies which gave similar results and revealed that the Na _v_1.8 channel was present in 95.0 ± 2.6% spermatozoa (n= 9, Figure 2A). In these experiments, the Na _v_1.8 signal was only measured in PI^+^ cells, to confirm that it derived exclusively from sperm cells.

Immunocytochemistry studies were performed using the three different Na _v_1.8 antibodies and showed the presence of the Na _v_1.8 protein in human spermatozoa (Figure 2B). In addition, these experiments demonstrated that the pattern of distribution of Na _v_1.8 immunoreactivity (IR) in sperm cells was similar in all tested samples. Positive IR was localized in the neck and along the flagellum in almost 100% of cells in all samples assayed (*n* = 9, 3 experiments performed with each antibody, Figure 2B). However, there were variations between cells in the presence and intensity of immunolabeling of different flagellar sections (Figure 2B). In approximately a 70% of cells in each preparation, the strongest immunolabeling was found in the principal piece while in other cells (approximately a 25%) the midpiece and the principal piece showed a similar staining intensity (Figure 2B). Approximately a 50% of cells showed an additional positive labeling in the sperm head, mostly in the acrosome and the equatorial segment, and, in a minor number of spermatozoa, Na _v_1.8 IR was also found in the post-acrosomal region (Figure 2B). Control assays incubated only with secondary antibodies showed no signal.

Western blot analyses of sperm homogenates were carried out using the three Na _v_1.8 antibodies. These studies confirmed the presence of the Na _v_1.8 protein in spermatozoa. The Na _v_1.8 antibodies recognized a single band with a molecular weight similar to the theoretical one (220 kDa). The results obtained with the mouse monoclonal antibody ab-93616 are shown in Figure 2C. The Na _v_1.8 immunoreactive band was not observed in control assays, where the primary antibody was omitted (not shown).

### Effects of veratridine on human sperm motility

**Figure 3:**
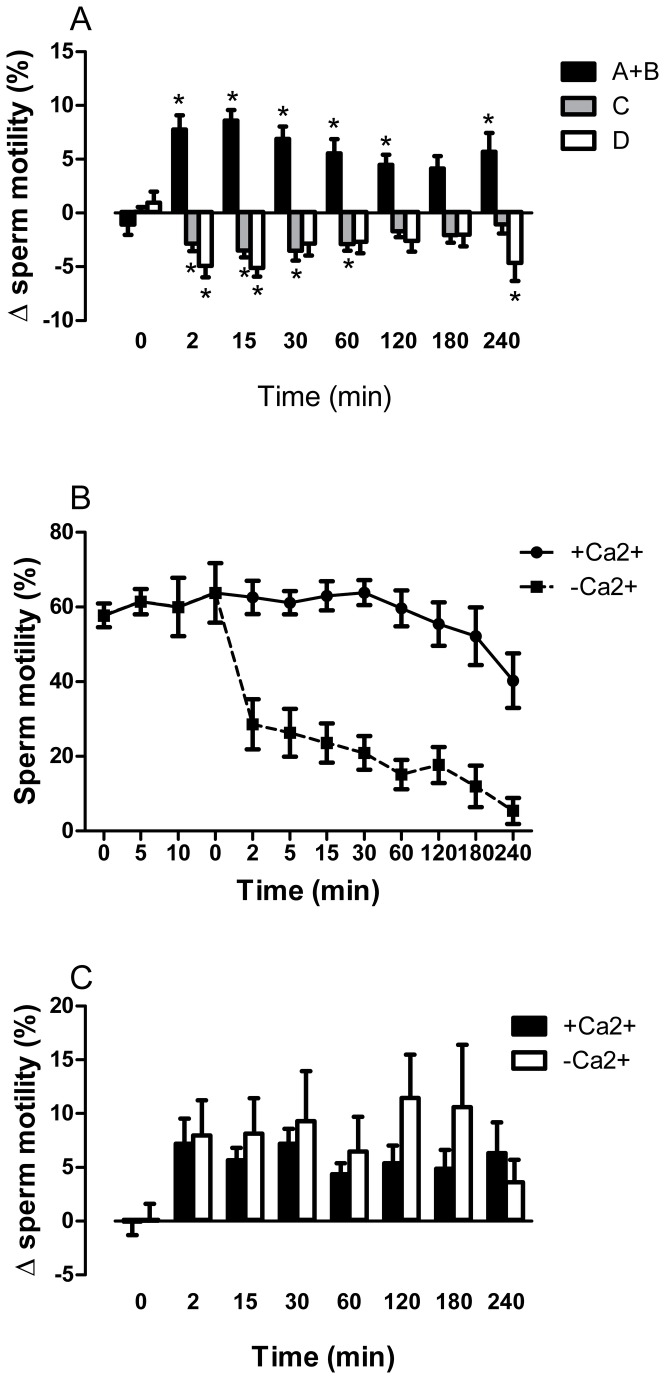
Effects of veratridine on human sperm motility in Ca^2+^-containing and Ca^2+^-free mHTF solution. (A) Effects of veratridine (10 μM) on progressive motility (grade A+B sperm), non-progressive motility (grade C sperm) and immotility (grade D sperm) at different times of incubation in Ca^2+^-containing mHTF solution. Bars are means with SEM of 36 different experiments and represent percentage changes in motility in samples treated with veratridine relative to the value observed at the same time in solvent-treated paired controls. *P<0.05, significant difference vs. control responses. (B) Time-dependent inactivation of sperm motility after incubation in a Ca^2+^-free mHTF solution. Afer capacitation for 2 h at 37°C in 5% CO_2_, the sperm suspension was divided in two aliquots, one of them incubated in Ca^2+^-containing solution and the other one in Ca^2+^-free solution. Data points are means with SEM of 7 different experiments and represent % motile sperm (grade A+B sperm). (C) Effects of veratridine (10 μM) at different times of incubation in Ca^2+^-containing and Ca^2+^-free mHTF solution. Bars are means with SEM of 7 different experiments and represent percentage changes in progressive motility (grade A+B sperm) relative to the value observed at the same time in solvent-treated paired controls.

In a previous report, we found that the VGSC activator veratridine (0.01-30 μM) caused concentration-dependent increases in human sperm progressive motility [18]. On the basis of these studies, the concentration of 10 μM was chosen for the present experiments. Veratridine (10 μM) caused rapid and maintained increases in progressive motility in capacitated sperm cells (Figure 3). The percentage of spermatozoa with progressive motility (A + B motility grade) increased immediately after veratridine addition and this effect was accompanied by a parallel decrease in the percentage of non-progressive mobile C grade and immotile D grade spermatozoa (Figure 3A). Veratridine-induced effects lasted for at least 4 hours and were observed in all sperm preparations assayed (*n* = 36, Figure 3).

Removal of Ca^2+^ from the bathing medium caused a rapid decrease in sperm motility followed by a slower gradual decrease in the percentage of motile sperm (Figure 3B). In this low Ca^2+^ medium, veratridine caused effects similar to those observed in normal Ca^2+^ mHTF medium, in terms of percentage increase in sperm motility (Figure 3C).

**Figure 4:**
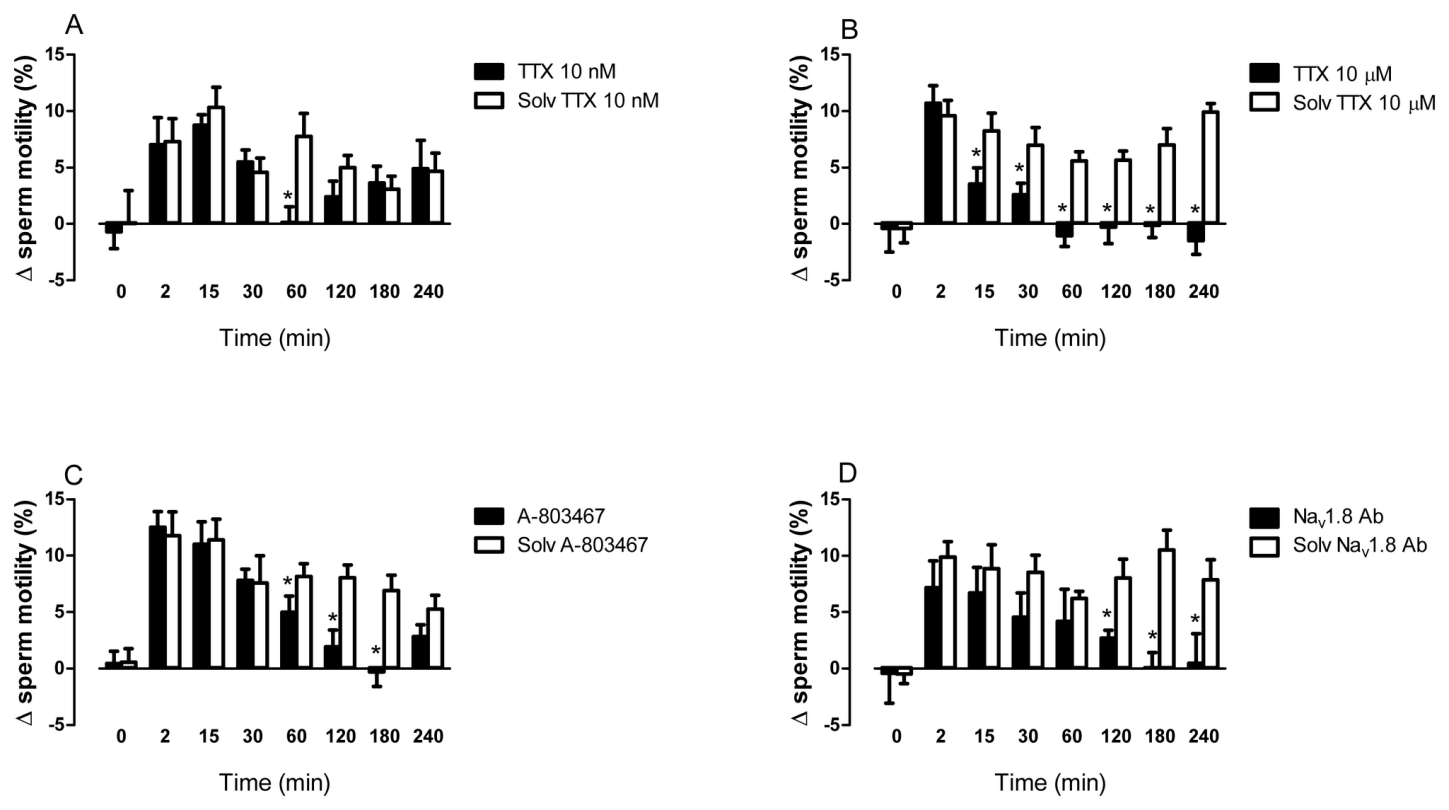
Effects of veratridine on human sperm motility in the presence of tetrodotoxin, A-803467 or ab-66743. The effects of veratridine (10 μM) after different incubation times were analyzed in the presence of (A) the VGSC inhibitor tetrodotoxin (TTX) (10 nM) (B) TTX (10 μM), (C) the selective Na _v_1.8 antagonist A-803467 (10 μM), (D) the Na _v_1.8 antibody ab-66743 (dilution 1:50) or the corresponding solvent. Bars are means with SEM of 6-8 different experiments and represent percentage changes in progressive motility (grade A+B sperm) relative to the value observed at the same time in the respective solvent-treated paired controls. *P<0.05, significant difference vs. responses to veratridine in the presence of the antagonist or antibody solvent.

The effect of veratridine on sperm motility was reduced in a time-dependent manner in sperm aliquots pretreated with the Na _v_1.8 antagonist A-803467 (1 μM) or with TTX (10 μM) or the Na _v_1.8 antibody ab-66743 (dilution 1:50) (Figure 4). The specific inhibitors A-803467 and Na _v_1.8 antibody mostly affected the late responses to veratridine (Figure 4). The corresponding solvents had no effect and did not modify the responses to veratridine in time-matched paired control aliquots (Figure 4).

Lower concentrations of TTX (10 nM), at which this compound acts selectively on TTX-sensitive VGSC, caused a punctual inhibition of the increases in motility observed 60 min after veratridine addition (Figure 4A).

TTX (10 μM), A-803467 (10 μM) or the Nav1.8 antibody ab-66743 (dilution 1:50) did not reduce basal sperm motility in untreated sperm samples (i.e., in the absence of veratridine or its solvent, data not shown).

### Effects of veratridine on hyperactivation and the acrosome reaction

**Figure 5:**
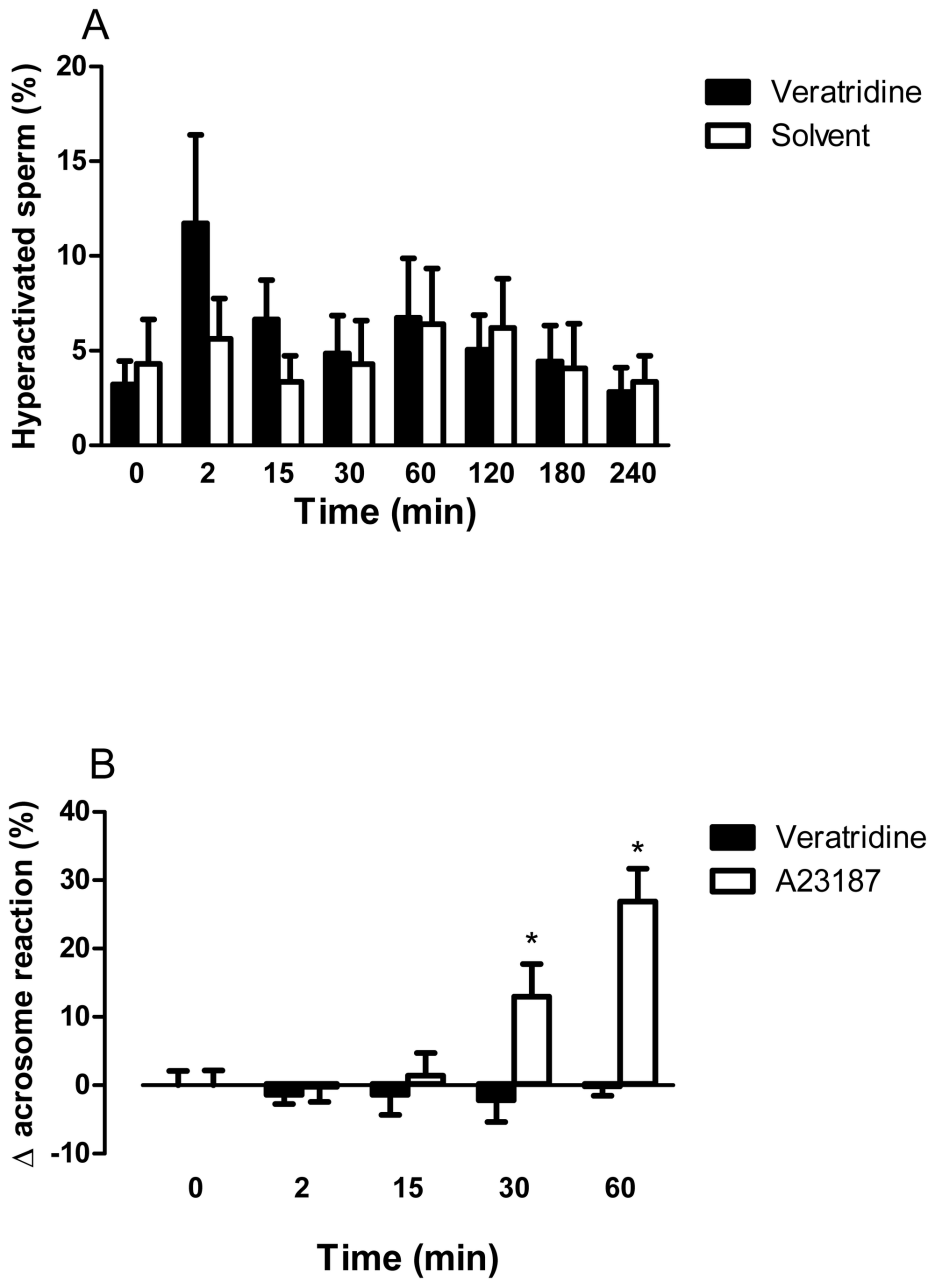
Time-dependent effects of veratridine on sperm hyperactivation and acrosome reaction (AR). (A) Effect of veratridine (10 μM) and its solvent on hyperactivated sperm motility. Capacitated sperm were treated with veratridine or its solvent for different times and hyperactivation evaluated by computer-assisted sperm analysis (CASA). Bars are means with SEM of 7 different experiments and represent % hyperactivated sperm. (B) Effect of veratridine and A-23187 on AR. Capacitated sperm were treated with veratridine (10 μM) or A-23187 (10 μM) for different times and the acrosomal status was assessed by staining sperm with FITC-PSA. Bars are means with SEM of 5 different experiments and were calculated as: (%AR reacted spermatozoa in veratridine- or A23187-treated aliquots) – (%AR reacted spermatozoa in the corresponding solvent-treated aliquots at the same time). ^*^ *P*<0.05, significant difference vs. values at time =0.

Spermatozoa were designated as hyperactive if they had a VCL > 150 μm/s; LIN < 50% and ALH > 7 μm. Figure 5A shows the values obtained in veratridine or solvent-treated samples, expressed as percentage hyperactive spermatozoa. Veratridine did not induce sperm hyperactivation after prolonged incubation (30-240 min). After incubation for 2 or 15 min, veratridine slightly increased hyperactivated sperm, although the increase did not reach statistical significance (*P*>0.05, *n*=7, Figure 5A).

Veratridine exposure for different times (2-60 min) failed to induce the AR in capacitated spermatozoa. Conversely, A23187 (10 μM) induced the AR in time-matched paired sperm aliquots (Figure 5). The spontaneous AR range in control, untreated aliquots was 8-12% and the solvent of A23187 and veratridine (DMSO), did not modify the AR status of the samples.

### Effects of veratridine on sperm intracellular free Ca^2+^ and Na^+^ concentrations

**Figure 6:**
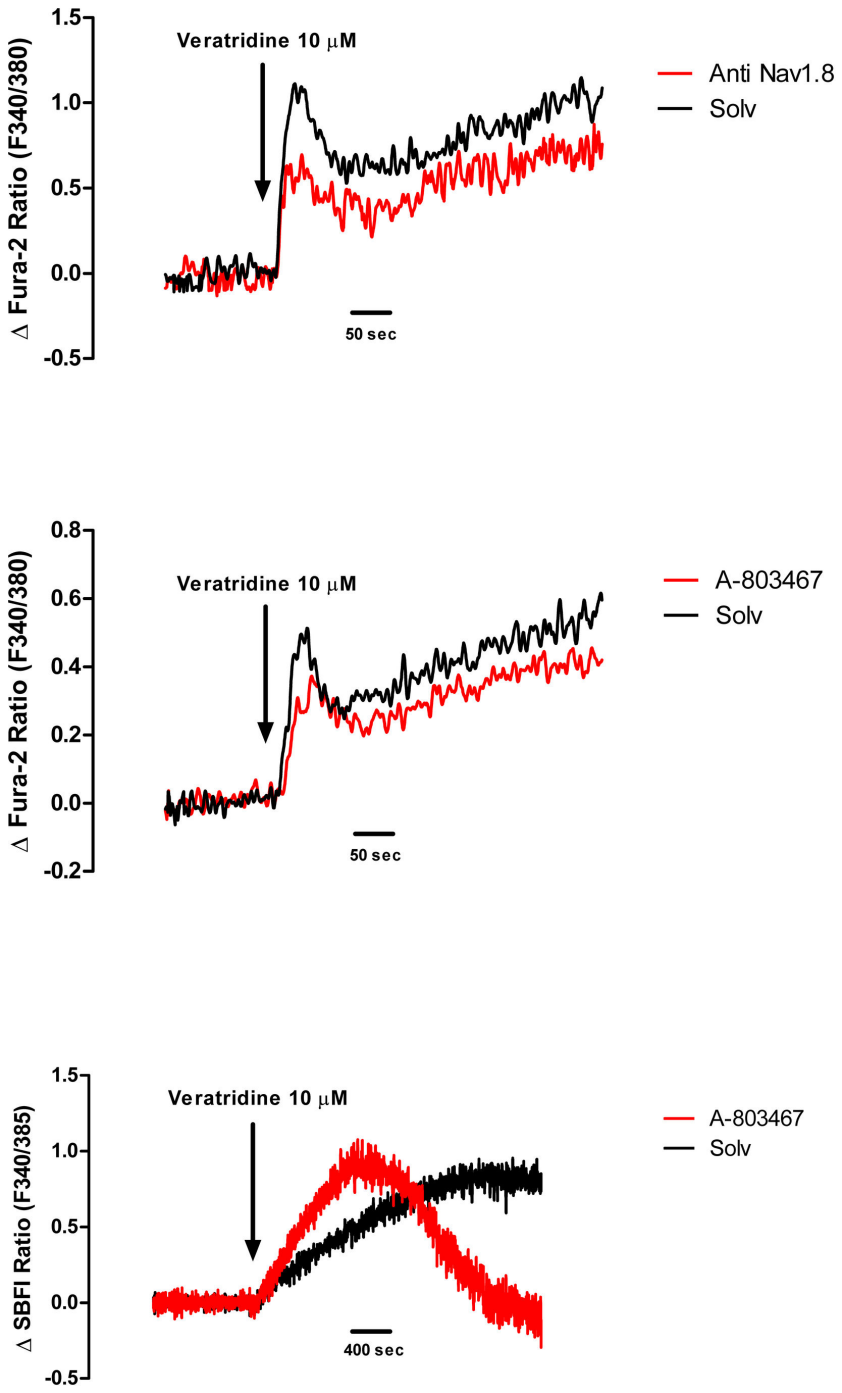
Effects of veratridine on intracellular free Ca^2+^ ([Ca^2+^]_i_) and intracellular free Na^+^ ([Na^+^]_i_) levels in human sperm cells. (A,B) For [Ca^2+^]_i_ measurement, cells were loaded with Fura-2 and responses to veratridine (10 μM) were determined in the presence of (A) the Na _v_1.8 antibody ab-66743 (dilution 1:50) (red line) or its solvent (black line) or (B) in the presence of the Na _v_1.8 antagonist A-803467 (1 μM) (red line) or its solvent (black line). The X axis shows time in seconds and the Y axis shows [Ca^2+^]_i_ data expressed by the F340/F380 ratio. Traces are representative of typical results obtained in 5-7 different experiments for each blocker. (C) For [Na^+^]_i_ measurement, cells were loaded with SBFI and responses to veratridine (10 μM) were determined in the presence of A-803467 (1 μM) (red line) or its solvent (black line). The X axis shows time in seconds and the Y axis shows [Na^+^]_i_ data expressed by the F340/F385 ratio. Traces are representative of typical results obtained in 6 different experiments.

In capacitated sperm suspensions loaded with the Ca^2+^ indicator Fura-2, veratridine (10 μM) caused a rapid small transient increase in [Ca^2+^]_i_ (Figure 5A) that declined slightly and was followed by a prolonged plateau that developed slowly (Figure 6A). The mean resting [Ca^2+^]_i_ was 112 ± 7 nM and raised to a maximal value of 146 ± 10 nM in the presence of veratridine (*n* = 43). In the same sperm aliquots, subsequent addition of progesterone caused the typical biphasic [Ca^2+^]_i_ signal consisting in a rapid transient response followed by a lower sustained [Ca^2+^]_i_ increase (not shown). The maximal [Ca^2+^]_i_ value reached in the presence of progesterone was 630 ± 91 nM. Thus, the maximal [Ca^2+^]_i_ increase produced by veratridine reached a 7.8 ± 0.7% of the maximal [Ca^2+^]_i_ response to progesterone (*n* = 43). The veratridine-induced [Ca^2+^]_i_ signal was not observed when spermatozoa were incubated in a Ca^2+^-free medium, demonstrating its dependence on extracellular Ca^2+^ influx.

The specific involvement of Na _v_1.8 was studied in paired sperm samples incubated for 6 h with the Na _v_1.8 antibody ab-66743 or its solvent (Figure 6A). The area of the veratridine-induced [Ca^2+^]_i_ signal measured during the 450 s next to its addition was slightly reduced in the presence of the Na _v_1.8 antibody and accounted for 79.2 ± 6.6% of the area measured in the presence of the solvent (*n* = 5, *P*<0.05). The area of the veratridine-induced [Ca^2+^]_i_ signal in the presence of A-803467 or TTX was 84.4 ± 4.0% (*n* = 5, *P*<0.05) and 92.7 ± 6.4% (*n* = 6, *P*>0.05) of the area measured in the presence of the solvent, respectively. With all the blockers, the small decrease of [Ca^2+^]_i_ was mainly due to a delay in the time to peak of the initial phasic response to veratridine (see Figure 6A,B).

Finally, the effect of veratridine on Na^+^ mobilization was investigated in capacitated sperm samples loaded with the fluorescent Na^+^ indicator SBFI. After veratridine addition, [Na^+^]_i_ increased in a progressive and sustained manner (Figure 6C). The [Na^+^]_i_ response to veratridine reached a 32.8 ± 9.3% of the maximal increase in [Na^+^]_i_ caused by the subsequent addition of EGTA. The veratridine-induced [Na^+^]_i_ signal was abolished in sperm cells incubated in Na^+^-free medium for 15 min before veratridine addition, demonstrating its dependence on Na^+^ entry from the extracellular medium. In the presence of A-803467, the increase in [Na^+^]_i_ caused by veratridine developed more rapidly, but the late sustained component of the response was abolished (see Figure 6C).

## Discussion

Ion transport through plasma membrane is a critical regulator in sperm physiology. Precise spatio-temporal changes in E_m_, pH_i_ and [Ca^2+^]_i_ contribute specially to the acquisition of fertilization competence in mammalian spermatozoa, including capacitation, hyperactivation, acrosome reaction and sperm-egg fusion [1-12,34,35]. However, the precise nature of these processes and the possible role of Na^+^ fluxes in spermatozoa remains incompletely understood.

Previously, we reported that several genes encoding voltage-gated Na^+^ channels were expressed in human testis and mature spermatozoa [18]. In this same context, the present findings show that the mRNA of one of these channels, Na _v_1.8, is abundantly expressed in human sperm and human testis. The expression of the *SCN10A* gene in human testis is consistent with the fact that transcription takes place in germ cells during the early stages of spermatogenesis, since sperm cells are considered as transcriptionally inactive. We used real-time qPCR to quantify the mRNA levels of *SCN10A* in 20 different human tissues and found that the human testis showed the highest expression. Thus, *SCN10A* expression was, in most tissues, more than 100-fold lower than that observed in the testis. The placenta, an organ that is devoid of nerves [36], showed the highest mRNA levels after the testis, providing evidence that, contrary to the extended idea [22,24], the Na _v_1.8 channel is also expressed in cells of non-neuronal origin and is particularly abundant in reproductive cells. Several Na _v_1.8 alternative splice variants have been identified [18] and, for this reason, we used two different primer pairs to demonstrate that the lack of *SCN10A* expression is not due to the presence of splice variants different from the wild type isoform in other tissues. These results strongly suggest that Na _v_1.8 can be considered as a testis- and sperm-specific Na^+^ channel and may play important, still undefined roles, in the regulation of male reproduction.

Flow cytometry and immunocytochemistry studies revealed that the Na _v_1.8 channel protein was abundantly expressed in human sperm and was present in the flagellum and around the neck in virtually all sperm cells in all preparations assayed. Na _v_1.8 was additionally localized in the equatorial segment or even in the acrosomal and post-acrosomal regions in a smaller population of spermatozoa within each sperm sample. The abundant expression of Na _v_1.8 in human sperm clearly argues for a role of this channel in the regulation of sperm function. Its predominant localization in the neck and the principal piece of the flagellum suggests that Na _v_1.8 could be involved in the modulation of flagellar activity and sperm motility, which is a critical parameter for sperm function and male fertility [13,37-40]. We reported previously that veratridine, a well-known activator of VGSCs, caused concentration-dependent increases of progressive motility in human sperm [18]. We thus analyzed whether the Na _v_1.8 could mediate the effects of veratridine on sperm motility. Because sperm cells are changing and their functional state varies with time, we examined the effect of veratridine on motility during a prolonged incubation time (4 h). Our results show that the effects of veratridine were inhibited in the presence of TTX at high (10 µM) concentrations, the selective Na _v_1.8 antagonist A-803467 and a specific anti-Na _v_1.8 antibody. These data provides functional evidence that the effects of veratridine on sperm motility involve activation of VGSCs and are mediated, in part, by activation of the Na _v_1.8 channel. Interestingly, neither TTX nor A-803467 or the anti-Na _v_1.8 antibody were able to reduce sperm resting motility in the absence of veratridine. These data strongly suggest that sperm VGSCs are activated by factor(s) exogenous to sperm or by a change in sperm surrounding conditions that are not reproduced in our *in vitro* studies. As indicated above, spermatozoa acquire their fertilization competence during their transit through the female genital tract. It may thus be hypothesized that such a factor or change could be produced in the female genital tract and, while increasing sperm progressive motility, may serve as a sense guide for sperm in their journey towards the oocyte.

A-803467 and the anti-Na _v_1.8 antibody inhibited the increases in motility produced after prolonged incubation with veratridine and did not affect the early responses to this ligand. This shows that the effects of veratridine on sperm are complex and involve activation of multiple mechanisms. In this context, our results show that TTX (10 μM) inhibited earlier responses to veratridine and TTX (10 nM) was able to reduce punctually the increases in motility observed 60 min after veratridine addition. Thus, veratridine may cause additional activation of still unidentified Na^+^ channels which are blocked by TTX. Our previous data showed that the TTX-sensitive VGSC Na _v_1.2, Na _v_1.4, Na _v_1.6 and Na _v_1.7 are present in human spermatozoa [18] and any of them could mediate the effects of TTX.

The early responses to veratridine appear to involve a slight activation of the sperm-specific cationic channel CatSper, which regulates [Ca^2+^]_i_ and is required for hyperactivation and the initiation of the acrosome reaction [3,7,8,12,14,15]. CatSper is promiscuously activated and mediates the effect on sperm of many different ligands [14]. In fact, we found that veratridine elicited a rapid small increase in [Ca^2+^]_i_ in mHTF medium and this effect was minimally affected by the VGSC blockers used in the present study. CatSper activation might also explain the slight hyperactivation observed after short exposure to veratridine. However, several experimental data suggest that the effects of veratridine on sperm motility are mainly independent on CatSper. First, veratridine failed to induce hyperactivation (except for the small early effect) and do not promote the AR (this study). Second, the effects of veratridine on motility are maintained in a Ca^2+^-free or in non-capacitating medium while, in these experimental conditions, the compound failed to induce any change in [Ca^2+^]_i_ ( [18] this study). Thus, veratridine appears to act mainly on the mechanism that mediates “activated” or “normal” progressive sperm motility. In this context, our data are in agreement with previous observations showing that deletion of the CatSper channel in mice does not influence normal motility and only affects sperm hyperactivation [15].

The participation of Na _v_1.8 in late responses to veratridine was further studied by analyzing the effect of the ligand on [Na^+^]_i_. Veratridine caused a delayed increase in [Na^+^]_i_, which is consistent with the observation that Na^+^ channel blockers mainly inhibited the late responses to veratridine. In the presence of A-803467, the veratridine-induced [Na^+^]_i_ signal developed more rapidly and the late sustained component of the [Na^+^]_i_ response was inhibited. The observation of a fast [Na^+^]_i_ increase, instead of a response inhibition, was unexpected. A possible explanation is that blockage of Na _v_1.8 permits the activation by veratridine of other Na^+^ channels present in sperm. In any case, these data again suggest that veratridine induces activation of multiple mechanisms and that Na _v_1.8 might play a role in mediating the late responses to veratridine in human sperm.

As indicated above, the CatSper channel plays a key role in the regulation of capacitation and hyperactivation, processes that occur in the oviduct [3,8,12,13]. This channel is activated by many different ligands including progesterone, the hormone that control sperm function within the female reproductive tract [1,2,8,15,35]. Progesterone is produced by cumulus cells surrounding the oocyte [1,12] but is found, although at lower concentrations, along the female reproductive tract [41]. Moreover, progesterone is able to activate potently the Catsper channel in human epididymal and testicular spermatozoa, i.e., previously to female insemination [15]. Thus, spermatozoa must have some mechanism(s) to prevent Catsper activation and sperm capacitation in a premature, inappropriate place. In this context, the present data show that veratridine has important effects on sperm motility, but only caused modest increases in [Ca^2+^]_i_ and did not induce hyperactivation or the acrosome reaction. Moreover, the effects of veratridine on sperm motility were mainly independent on external Ca^2+^ and on the capacitated or noncapacitated state of sperm ( [18] this study) suggesting that they did not require activation of Catsper. On the other hand, the present results strongly argues for a participation of the Na _v_1.8 channel in mediating, in part, the effect of veratridine on [Na^+^]_i_ and normal sperm motility. It has recently been reported that capacitation of human sperm is accompanied by a reduction in the activity of Na^+^ channels [42] and a decrease of [Na^+^]_i_ [43]. Thus, activation of VGSCs, including Na _v_1.8, and, probably also, epithelial Na^+^ channels of the ENaC family [17,42] could be a mechanism that facilitate sperm swimming in the long travel throughout the female reproductive tract while avoiding premature sperm capacitation.

In conclusion, this study shows that the Na _v_1.8 channel is abundantly and specifically expressed in human testis and sperm and suggests that this voltage-gated Na^+^ channel plays a role in the regulation of normal sperm motility or in the fine tuning of this activity. As it occurs with many other channels present in ejaculated spermatozoa [1], Na _v_1.8-null mice are fertile [22]. This could suggest that these channels have a minor role, if any, in the regulation of sperm function. Alternatively, the presence of a great variety of channels could allow for function compensation, in the case of failure of a particular channel [1]. The later could explain the complex effects exerted by veratridine and the Na _v_1.8 channel blockers on sperm function. Additional studies will help to clarify more precisely the role of Na _v_1.8 in sperm function and male fertility.

## Supporting Information

Figure S1 **Analysis of human sperm capacitation status by investigation of sperm protein tyrosine phosphorylation.** Following isolation of spermatozoa by centrifugation through a Percoll discontinuous gradient and swim-up, sperm were incubated in mHTF medium supplemented with 10 mM HEPES and 0.5% BSA. Sperm aliquots were removed at different time periods and sperm proteins were analyzed for Tyr phosphorylation by Western blotting using the monoclonal antiphosphotyrosine antibody PY20. Lane 1, 0 h at room temperature; lane 2, plus 2 h at room temperature; lane 3, plus 1 h at 37°C in 5% CO_2_; lane 4, plus 2 h at 37°C in 5% CO_2_; lane 5, plus 4 h at 37°C in 5% CO_2_. The figure shows a typical experiment and is representative of results obtained in 6 different samples.
(TIF)
